# Non-Covalent Derivatives: Cocrystals and Eutectics

**DOI:** 10.3390/molecules200814833

**Published:** 2015-08-14

**Authors:** Emily Stoler, John C. Warner

**Affiliations:** The Warner Babcock Institute for Green Chemistry, 100 Research Drive, Wilmington, MA 01887, USA; E-Mail: emily.stoler@warnerbabcock.com

**Keywords:** non-covalent derivative, cocrystals, eutectics, green chemistry

## Abstract

Non-covalent derivatives (NCDs) are formed by incorporating one (or more) coformer molecule(s) into the matrix of a parent molecule via non-covalent forces. These forces can include ionic forces, Van der Waals forces, hydrogen bonding, lipophilic-lipophilic interactions and pi-pi interactions. NCDs, in both cocrystal and eutectic forms, possess properties that are unique to their supramolecular matrix. These properties include critical product performance factors such as solubility, stability and bioavailability. NCDs have been used to tailor materials for a variety of applications and have the potential to be used in an even broader range of materials and processes. NCDs can be prepared using little or no solvent and none of the reagents typical to synthetic modifications. Thus, NCDs represent a powerfully versatile, environmentally-friendly and cost-effective opportunity.

## 1. Introduction

A non-covalent derivative (NCD) [1] is formed when one (or more) coformer molecule(s) is incorporated into the matrix of the target molecule by way of non-covalent forces [2,3,4]. These forces can include ionic and Van der Waals forces, hydrogen bonding, lipophilic-lipophilic interactions and pi-pi interactions. The NCDs self-assemble according to mutual molecular recognition based on the co-formers’ cooperative topography.

NCDs often show significant differences when compared to their parent molecules. These differences can include dramatic changes in solubility, melting point, optical properties, bioavailability, or stability. In the founding work on green chemistry, *Green Chemistry: Theory and Practice*, NCDs were recognized as a viable and powerful addition to traditional synthetic derivatization methodologies for altering a material’s physical properties [5]. In light of this powerful utility and the fact that NCDs can be formed by way of economic and environmentally-friendly means, NCD-based strategies can have significant value in the development of greener chemistries.

Eutectics and cocrystals are material forms that both fall under the umbrella of NCDs. The definition of a cocrystal has developed, after initial debate [6,7,8], as a homogenous crystalline material that is made up of two or more molecules in definite stoichiometric amounts held together by non-covalent forces [9]. A cocrystal is distinguishable from a salt by the degree of proton sharing [10]. According to the *FDA’s* guideline for the classification of cocrystals [11], a cocrystal is required to have a pKa difference between coformers that is less than 1, thus indicating a non-ionic species and minimal proton sharing. This is a limiting definition since cocrystals rather than salts can occur at pKa differences as large as 4 [12]. A cocrystal is also differentiated by the state of matter of its starting materials. If both the starting materials are solid then the solid NCD product is a cocrystal. If one of the combining materials is a liquid, then the resulting combined substance is termed a solvate [13]. A eutectic is also formed from two solids, but the resulting NCD is a homogenous phase formed at the point, both with respect to temperature and molar ratio, at which the two solids become perfectly miscible and is recognizable as the minimum coherent point on the phase diagram.

Cocrystals exhibit short-range order, *i.e*., an order that exists between neighboring atoms. This same short-range order likely also exists in eutectics [14,15]. In both crystals and eutectics, this short-range order could be associated with a preference for heteromeric *vs.* homomeric molecular interactions at the relevant molecular ratios. For example, the neutron diffraction patterns of Au-Si eutectics point to the preferred association of unlike atoms [16] and are consistent with similar conclusions drawn from the thermodynamic work of Chen and Turnbull [17]. On the continuum of long-range order, however, eutectics and cocrystals occupy opposite positions. A crystal is defined by its long-range order. In contrast, the eutectic lacks long-range order.

Whether a cocrystal or a eutectic will result from a non-covalent derivatization depends on the predominant thermodynamic force. A cocrystal will result when the enthalpic advantage outweighs any entropy loss due to ordering. On the other hand, the eutectic will predominate when the entropy gain outweighs the potential enthalpy gain of an ordered crystal. The conditions for this entropic *vs.* enthalpic control can be attributed in part to the parent molecules’ topography. In cases where the functional groups are compatible for effective non-covalent bonding and the size and shape of the parent molecules favor crystal packing, a cocrystal will result. In cases where the non-covalent bonding is favored but there is little or no packing shape compatibility of the parent molecules, then the binding forces predominate and a eutectic is produced [18].

## 2. Preparation of NCDs

Compared to traditional synthetic modifications, the preparation of NCDs is an alternative that can be less toxic, produce less waste, be less labor-intensive and more economical when used to affect the same types of property changes [3]. A variety of solvent methods can be used to prepare NCDs. Cocrystal preparation has been reported using traditional methods of precipitation [19,20,21,22], cooling crystallization [23,24] and slurry formation [25]. Evaporation [26,27,28,29,30,31,32,33] has emerged as the most common solvent-based method for the preparation of cocrystals [34]. The preparation of eutectics has also been reported using solvent evaporation [35,36]. Solvent-based preparation methods for NCDs have significant limitations. All the NCD partners are required to have a solubility relationship with the solvent that allows for effective dissolution followed by concurrent precipitation as the solution cools or evaporates.

As Etter’s early hydrogen-bonded cocrystal work aptly demonstrated [30] and later hydroquinone work supported [2], solid-state grinding is a viable alternative to solvent methods. Since those first works, solid-state grinding has become a well-established method for the preparation of NCDs. A large number of examples can be found in the literature describing the grinding of neat solids via mortar and pestle or automated grinders to produce cocrystals [20,37,38,39,40] and eutectics [41,42]. There are three mechanisms recognized to be in effect during grinding in the solid state: grind molecular diffusion, eutectic formation, and cocrystallization mediated by amorphous phase [43]. Grind molecular diffusion is described as a process by which the surface of the solid is made mobile either by vaporization or energy transfer to the surface of the solids. In the case of the eutectic, the eutectic liquid is first formed which then crystallizes to the final solid. Lastly, the cocrystal is described as coalescing out of the amorphous phase of the parent solids. In each of these cases, the common feature is that of forming an intermediate phase (gas, liquid or amorphous solid) that has a higher mobility and/or energy with respect to starting materials [44].

The addition of catalytic amounts of solvent to the grinding matrix has been shown to improve the efficiency of preparation [44] and increase the crystallinity of the final cocrystal product [45]. These observations and subsequent studies [43] have led to the common practice of liquid-assisted grinding (LAG), originally referred to as solvent drop grinding, for the preparation of NCDs [46,47,48]. In a study comparing method preparations, it was demonstrated that it was possible to prepare the same carbamazepine cocrystals achieved with evaporation by means of solvent-assisted grinding [49]. In addition, grinding can produce cocrystals that are not available by solvent-based methods [20,50].

The spray-dry evaporation technique has also been used to produce different cocrystal forms from the same parent compounds [51]. Cocrystals of urea and succinic acid when prepared with spray drying produced a previously unidentified cocrystal structure, having a 1-to-1 molar ratio and a laminar sheet form. These same materials, with other preparation methods, produced a 2-to-1 urea-to-succinic acid structure with a complex three-dimensional structure that contains two ring-structure repeat units. The two cocrystal forms had different stabilities and dissolution rates. This study is indicative of the versatility of NCD formation, not only in choice of materials, but also in the choice of method in order to produce tailored NCDs.

The practical industrial manufacture of cocrystals has yet to be fully exploited because the most common methods for the preparation of cocrystals, solid-state grinding or slow evaporation, do not lend themselves easily to scaling up. However, some limited promise was seen in the case of carbamazepine/saccharin in which the cocrystal was prepared at a 30 g scale by solution crystallization [19]. Several references discuss large batch cooling crystallization for the preparation of cocrystals facilitated by the preparation of ternary phase diagrams for the interaction of coformers and the solvent [52,53]. In another promising example, the formation of carbamazepine (CBZ)-trans-cinnamic acid cocrystals, in stoichiometric ratios, was reported using continuous hot melt extrusion [54]. In a similar set of studies, CBZ-nicotinamide cocrystals were prepared in the presence of a polymer matrix using hot melt extrusion [55]. Another interesting option for large scale production is demonstrated through the preparation of cocrystals through vapor deposition of triiodotrifluorobenzene onto a layer of 1,4-bis-(*E*)-2-(pyridin-4-yl) vinyl benzene [56].

## 3. Characterization of NCDs

A wide variety of single crystal structures of cocrystals can be found in the literature [26,57,58,59,60,61,62]. This crystal structure analysis is the most direct and conclusive method for NCD characterization. However, a cocrystal of the appropriate size for single crystal analysis is oftentimes not achievable, particularly when grinding is used for preparation. In these cases, powder X-ray diffraction (PXRD) has been used as an alternative characterization technique. Indexing software was used to identify the crystal structures from the PXRD data of theobromine cocrystals with either trifluoroacetic acid or malonic acid [63]. Because PXRD alone cannot differentiate between hydrates, solvates, and cocrystals, the data is often considered in conjunction with another analysis method [64]. For example, by combining PXRD, nuclear magnetic resonance (NMR) and theoretical calculations, theophylline-nicotinamide cocrystal structures were solved from the powder cocrystal data [65].

In addition to solid-state NMR (SS-NMR), other spectroscopic methods are frequently employed for the identification of non-covalent derivatives. These methods are not generally predictive of the structure of the cocrystal or eutectic, but can, by the appearance of novel peaks in the spectra, indicate the presence of novel interactions in the NCD that are not seen in the pure parent compounds. In one example, terahertz spectroscopy was used to follow the mechanochemical construction of a two-component cocrystal that was made by grinding together phenazine and mesaconic acid [38]. In some cases, especially those where the coformer is a carboxylic acid, infrared (IR) spectroscopy can be used because the frequency of some stretches will change when in a cocrystal environment [66]. Raman spectroscopy has also proven to be an effective tool for NCD analysis as exemplified by the analysis of cocrystals prepared from salicylic acid and a series of coformers [67].

Differential scanning calorimetry (DSC) can be used to identify the melting point of NCDs. Additionally, phase diagrams developed by plotting the mole percent of the coformer against the melting point changes can be powerful tools for differentiating between the NCD’s cocrystal and eutectic forms [68]. DSC thermal data for eutectic mixtures results in a classic V shape where the minimum point of the V represents the molar ratio and temperature at the eutectic point. By contrast, the binary-phase diagram for a cocrystal exhibits a more complex behavior which contains two eutectic points and a region of cocrystal at the maximum between the two eutectic points. This results in a typical W-shaped phase diagram for cocrystals [69,70,71].

Microscopy has also been used to indicate the formation of a cocrystal, though this method can only indicate a significant change in morphology. In one example, the interface of cocrystal preparation for lamotrigine and phthalimide was observed by hot-stage microscopy [72]. In another study, atomic force microscopy (AFM) was used to differentiate between two forms of caffeine-glutaric acid cocrystals [73].

## 4. Applications of NCDs

Because of their versatility, NCDs have useful applications in a wide variety of areas. In the early 1990s, one of the earliest industrial patents with NCDs demonstrates the use of non-covalent derivatives applied to the development process of instant film [74]. NCDs were used to alleviate the problem of labile hydroquinone that leads to premature aging and diffusion between photographic film layers. Based on the naturally occurring quinhydrone NCD, cocrystals between hydroquinone and diamides were used to reduce the hydroquinone solubility and diffusion. A substituted hydroquinone was also prepared with diamides which were shown to increase the hydroquinone’s stability toward oxidation when in the form of emulsions in water [75]. This early NCD work also included a series of studies exploring the hydroquinone NCD system via changes to the coformer structure [2,76,77]. Even relatively minor changes in the coformer structure have significant effect. For example, hydroquinone coordinated with bis(*N*,*N*-diethyl)terephthalamide was reported to form a 1:1 complex with a melting point of 147 °C. The bis(*N*,*N*-dimethyl) analogue of the amide, however, resulted in a 2:1 hydroquinone-to-amide complex with a melting point of 198 °C [2]. In a similar example, hydroquinone and tris-(*N*,*N*-diethyl)trimesamides formed a 3:2 complex while tris-(*N*,*N*-dimethyl)trimesamides formed a complex of 2:1 hydroquione to trimesamide [76]. These studies speak to the enormous capacity to tailor NCD systems for specific properties.

### Pharmaceuticals

Much attention has shifted to the potential utility of cocrystals in the pharmaceutical industry. A variety of active pharmaceutical ingredients (APIs) have been incorporated into cocrystals and eutectics, with the result being remarkable changes in physical properties without the loss of pharmaceutical activity. Improvements to solubility [78,79,80,81,82,83] and hydro-stability [20] have been reported for cocrystals with APIs. Dovetailing with the changes in solubility, preparation of pharmaceuticals as cocrystals has been hailed as one of the most effective methods available for the challenging task of improving bioavailability [84]. A number of review articles are in print on pharmaceutical NCD cocrystals [13,66,85,86,87,88]. Likewise, pharmaceutical eutectics have been recognized as a powerful means for altering API properties [88].

In an illustrative and often referenced study, several cocrystals were prepared from fluoxetine hydrochloride and the solubility was measured. The fluoexetine hydrochloride solubility was determined to be 11.6 mg/mL. The benzoic acid cocrystal solubility was measured at 5.6 mg/mL. Both the fumaric acid and succinic acid cocrystals were found to increase the solubility to 14.8 mg/mL and 20.2 mg/mL, respectively [31]. In a similar study, the cocrystal formed from the chemotheraphy agent, Tegafure, was shown to have solubility comparable to that of Tegafure in its pure amorphous phase and much higher than in its pure crystalline phase. The advantage of the cocrystal in this case was the improvement in solubility without the loss of stability associated with the amorphous phase [86]. A number of similar examples showing changes in solubility and dissolution for cocrystals can be found in the literature and several reviews have been published on the subject [87,88,89,90]. The literature on eutectics and API solubility is less plentiful, but in one example the 1:1 binary eutectic formed from two anti-tubercular drugs, pyrazinamide and isoniazid, showed significantly improved intrinsic dissolution [42]. In another example PEG and a variety of APIs were prepared as eutectics to provide enhanced API solubility [91]. Ibuprofen-menthol eutectics for use in suppositories also showed improved solubility over pure ibuprofen [92].

This change in solubility has implied effects on the bioavailability of APIs. This can be seen in glutaric acid cocrystals prepared with the API, 2-[4-(4-chloro-2-fluorophenoxy)phenyl] pyrimidine-4-carboxamide. The cocrystals showed an increase in aqueous dissolution and showed an improved bioavailability when compared to the pure API [24]. In a similar example, 1:1 danazol:vanillin cocrystals were prepared that showed improved bioavailability by comparison to the poorly available pure danazol [81]. Stability enhancement has also been reported with cocrystals [93]. Cocrystals prepared with carbamazepine showed a higher resistance to hydrate formation [94]. Superior humidity stability was reported for cocrystals of theophylline and oxalic acid.

## 5. Cosmetics

Typical cosmetic formulations involve preparing the active ingredient in a format that is both easy to apply and reasonably stable. Eutectic mixtures and cocrystals have been widely reported to facilitate these preparations. Some solid fragrances are a challenge to include in formulations because the high temperatures necessary to melt the solids result in degradation of the other formulation components. Eutectic mixtures of standard solid fragrances with benzophenone were prepared that resulted in liquids that could be readily incorporated into formulations. The versatility and tailorability of these types of NCDs was demonstrated by the range of eutectic mixtures prepared from solid fragrances and benzoquinone [95]. In a similar example, eutectic mixtures were prepared from 12-hydroxystearic acid. While 12-hydroxystearic acid itself has well-known benefits to the skin, it is a solid with a high melting point and limited bioavailability. The eutectic mixtures provided lower-melting-point solids that could provide more availability [96]. The UVB absorber butyl methoxydibenzoylmethane (BMDM) was incorporated into a eutectic with or without the 12-hydroxystearic acid to produce liquids that overcome the challenge of the high melting point of both [97]. Eutectic mixtures were used another anti-sun formulation wherein mixtures of *n*-butylphthalamide and isopropylphthalamide were prepared with 1,3,5-triazine derivatives resulting in unusual stability [98]. A eutectic mixture was used in scalp itch treatment formulations [99,100] that showed improved deposition of the eutectic mixtures of monoethanolamides on the scalp *vs.* the pure amides.

Cocrystals of 3-iodopropynyl butylcarbamate, an antifungal agent used in personal care products, were reported [101]. The cocrystal compositions were reported to have better physical and chemical properties, in particular greater solubility in water and greater heat stability. The cocrystal compositions were also reported to have better processability properties such as better powder flowability and better compressibility for tablet formation. Cocrystals of *p*-coumaric acid and nicotinamide were reported as preparations for acne treatment, though no additional performance parameters were reported [102]. In an example coming from our labs, it was shown that NCDs prepared from the hair dye colorant are more stable on the hair than the pure colorant [103,104].

## 6. Agrochemicals

Numerous patents for cocrystals used for agricultural products, such as fungicides, fertilizers and insecticides have been filed. For example, cocrystals of the fungicide metalaxyl were prepared with a second broad spectrum fungicide prothioconazole, to produce a material with dramatically decreased water solubility *vs.* that of pure metalaxyl [105]. The advantage in the decreased water solubility was described as serving to reduce the run-off of the fungicides and thus limit the amount of fungicides necessary for efficacy as well as preventing excessive run-off into ground water streams. This is a particularly interesting example because both partners in the cocrystal are active ingredients and are working to form a mutually beneficial product.

Cocrystals of the herbicide 3,6-dichloro-2-methoxybenzoic acid were prepared with a variety of nitrogen containing heterocycles [106]. These cocrystals were reported to address not only the issue of excessive water solubility but also that of stability. In particular, some herbicides were reported to suffer from the Ostwald effect over time, *i.e*., the growth of large crystals during the aging process. These large crystals are considered to have a deleterious effect during both the processability of the material during production and on the efficacy of the herbicide during use. The cocrystals were reported to have improved stability against this aging process. 4-hydroxybenzoic acid was reported to be effective with numerous pesticides and herbicides when prepared as a cocrystal [107].

Cocrystals were also used to raise the melting point of an imidacloprid insecticide using oxalic acid [108]. The raised melting point allowed for better shelf stability and prevented the melting and clumping of the pure insecticide with aging. The same group published a similar patent reporting increased stability with raised melting point of 4-{[(6-chloropyrid-3-yl) methyl](2,2-difluoroethyl)amino}furan-2(5*H*)-one with salicylic acid [109].

## 7. Chromophores

Pigments are a particularly interesting chromophore application of NCDs. Bucar *et al*. make a compelling argument for the use of mechanochemical grinding to create novel pigments in quantitative yields that would otherwise be unachievable by solvent methods. The effective preparation of three color-tuned fluorescein cocrystals were used as model studies to support this argument [37]. Cocrystals of titanyl fluorothalocyanine with titanyl fluorocyanine are described [110] and prepared by heating [111] and dry milling [112]. The resulting cocrystals not only have a novel spectrum but, more importantly, also have improved electrophotographic sensitivity and low dark decay. Red textile pigments were prepared as two-component diazo-based eutectics. These pigments were shown to have performance equal to those of more toxic dyes in terms of color fastness, heat resistance, acid resistance, alkali resistance and solubility [113]. Yan *et al.* demonstrated that cocrystals can be used for more than tuning the color, stability and solubility of chromophores. They prepared a series of cocrystals of stilbene-type molecules with different co-formers that show remarkable differences in terms of luminescence emissions, UV/Vis absorbance and quantum yield [114].

## 8. Food Additives

NCDs have also found a place in food additives. Cocrystals prepared from the antioxidant yerba mate and sucrose resulted in powders with lowered hygroscopicity and good flowability for processing when compared to yerba mate alone [115]. It was also confirmed that yerba mate did not lose its antioxidant activity in the cocrystal form. Cocrystals were also used to solve another processing problem. While the mixture of ethylvanilla and vanilla is desirable from a taste and fragrance perspective, the simple mixture produces clumping that makes it unusable in a manufacturing process. Cocrystals prepared from each of these compounds prior to their combination produced powders that had good flow and therefore good potential for use in the food and fragrance industry [116]. Cocrystals of menthol and xylitol were prepared as another flavor additive with better processing properties. The cocrystals were reported to show lower hygroscopicity compared with xylitol and a higher solubility in water compared with menthol [117].

## 9. Other Potential Applications

NCDs have enormous commercial potential for other less-developed applications as well. Despite limited patent activity, they are worth mentioning because the effects are notable. Cocrystals have been used to alter electrical properties and shown to have potential as organic semi-conductors. It was shown that cocrystallization resulted in a material that is more electrically conductive than the parent molecule [118]. This is an area of great interest for power production and should be further explored.

Cocrystal reagents are also an interesting opportunity. Solid-state reactions were run in high yield by preparing cocrystals from olefins. The indications are that it is possible to use cocrystals to direct reactivity [119]. In one example, unique photochemical reactions of diarylethene were made possible by the conformation induced by the cocrystal [120]. Cocrystals were also used to effect chiral resolution. Cocrystals were prepared from the racemic mixture DL-arginine. The difference in solubility between the D and L based cocrystals results in enantiomeric separation [121].

## 10. Summary

NCDs, encompassing both cocrystal and eutectic forms, possess properties that are unique to their supramolecular matrix. These properties include critical product performance factors such as solubility, stability and bioavailability. NCDs have been used to tailor materials for a variety of applications and have the potential to be valuable in an even broader range of materials and processes. NCDs can be prepared using little or no solvent and none of the reagents typical to synthetic modifications. Thus, non-covalent derivatization is a powerfully versatile, environmentally-friendly and cost-effective tool.
